# More often than not, we’re in sync: patient and caregiver well-being over time in stem cell transplantation

**DOI:** 10.1186/s12955-021-01909-3

**Published:** 2022-01-10

**Authors:** Timothy S. Sannes, Krista W. Ranby, Miryam Yusufov, Benjamin W. Brewer, Jamie M. Jacobs, Stephanie Callan, Gillian R. Ulrich, Nicole A. Pensak, Crystal Natvig, Mark L. Laudenslager

**Affiliations:** 1 Department of Psychiatry, University of Colorado Anschutz Medical Campus, Aurora, USA; 2 Division of Adult Psychosocial Oncology and Palliative Care, Dana Farber Cancer Institute, Harvard Medical School, Boston, MA USA; 3 Department of Psychology, The University of Colorado Denver, Denver, USA; 4 School of Medicine Anschutz Medical Campus, Division of Hematology, The University of Colorado, Aurora, USA; 5 Department of Psychiatry, Harvard Medical School, Massachusetts General Hospital, Boston, USA; 6 Redbank Anxiety Therapy, Little Silver, NJ USA

**Keywords:** Caregiving, Dyads, Stem Cell Transplantation, Quality of Life, Anxiety, Depression

## Abstract

**Background:**

Hematopoietic stem cell transplantation (HSCT) is an aggressive medical procedure which significantly impacts the shared emotional well-being of patients and family caregivers (FC). Prior work has highlighted the significant overlap in well-being among patients and FCs; however, how this interdependence may change over the course of HSCT has received less attention.

**Methods:**

We conducted secondary analyses of a supportive intervention delivered to 154 FCs of HSCT patients and examined relationships at baseline, 6 weeks, 3 and 6 months post-HSCT. Actor Partner Interdependence Modeling examined patient quality of life (QOL) and FC anxiety/depression.

**Results:**

The data did not fit a multigroup approach limiting our ability to test intervention effects; however, bivariate analyses indicated FC depression significantly correlated to patient QOL at baseline (*r* = − .32), 6 weeks (*r* = − .22) and 6 months post-HSCT (*r* = − .34; *p*’s < .05); whereas FC anxiety was only correlated with patient QOL at the first two timepoints (*p*’s < .05). There was an unexpected, partner effect such that worse patient QOL at 6-weeks significantly related to *lower* FC depression at 3-months (*B* = .193; *p* = .026) and changed direction with patient QOL at 3-months being related to more FC depression at 6-months (*B* = − .187; *p* = .001).

**Conclusions:**

These findings highlight the significant, yet nuanced, interdependence of patient QOL and FC well-being during HSCT. Specifically, greater interdependence was observed between patient QOL and FC depression compared to FC anxiety, suggesting potential treatment targets for patients and their families.

Trial was registered at ClinicalTrials.gov Identifier: NCT02037568; first registered: January 16, 2014; https://clinicaltrials.gov/ct2/show/NCT02037568

## Background

Cancer impacts not only patients, but also their family members and informal caregivers. There are approximately 3.2 million unpaid family cancer caregivers in the United States today [1]. The number of caregivers needed to support cancer patients continues to rise [2]. As cancer treatment at large moves toward the outpatient setting, caregivers will be expected to provide increasing levels of unpaid care [3], potentially contributing to the already known physical and psychological morbidity associated with caregiving [4]. Caregiving for cancer patients can increase risk for caregivers’ social isolation, sleep problems [5], loneliness [6], depression, and anxiety [7].

The emotional toll of cancer caregiving can have detrimental effects on both caregivers and their patients. Indeed, a large body of evidence suggests that caregivers’ and their patients’ psychological functioning are inextricably linked [8, 9] such that when caregivers become more depressed, patients are likely to experience similar depressive symptoms, and vice versa. This reciprocity is often termed interdependence [10] or, in more stark terms, an emotional contagion [11]. Thus, there is strong empirical evidence that patient-caregiver dyads overlap in their emotional experience, supporting the theoretical basis for examining dyadic interdependence. While there is agreement that this interdependence occurs over the course of cancer treatment, we know much less about how and when interdependence within patient-caregiver dyads may change over time [12].

Psychological interdependence among patient-caregiver dyads is particularly important during hematopoietic stem cell transplant (HSCT). HSCT is an aggressive medical procedure in which patients’ bone marrow is ablated with chemotherapy, with or without radiation, donor cells are carefully matched to the patients and then infused into the patient, and the medical team supports the patient while the immune system is reconstituted over the next year or more [13]. For patients, emotional distress typically peaks leading into HSCT, and symptomatology continues to increase through the course of transplant during hospitalization. Patients’ anxiety and depression begin to decrease 3 and 6 months following HSCT [14]. For caregivers, distress is also high leading up to HSCT, with depression and anxiety increasing during the hospitalization and course of transplant [15]. Caregivers’ physical and emotion well-being can deteriorate in the months following transplant [16].

Despite the interdependent nature of patients and caregivers’ well-being during the HSCT process, relatively little work has examined this dynamic over time [12]. For the current study, we sought to address this gap by drawing from patients undergoing HSCT in the context of a stress management intervention targeting the caregiver. The intervention, called PEPRR (PsychoEducation, Paced Respiration, and Relaxation) [17], was effective at reducing caregiver distress [18], yet dyads’ interdependence over time was not investigated. Thus, our goal was to examine the potential interdependence of caregiver well-being and patient quality of life and, potentially, how the PEPPR intervention impacted dyad’s shared well-being during HSCT. In line with other dyadic research in HSCT populations [19] as well as our work with this study’s baseline data [20], we hypothesized that the emotional well-being of patients and caregivers would demonstrate a stronger interdependence over time in the PEPPR group, as caregivers may attend to their own emotional needs, thereby growing the emotional bond within the dyad.

## Methods

These secondary data analyses drew from a 1:1 randomized clinical trial in which HSCT patients and their caregivers were randomly assigned to either caregivers receiving PEPPR or enhanced treatment as usual (eTAU; intervention materials were provided without prompting or coaching) [18]. The 4 study timepoints were anchored to patients’ day zero of HSCT (baseline) at which point questionnaire data was collected, as well as at 6 weeks, 3 months and 6 months post-transplant. Patient and caregivers completed the measures described below at these timepoints with baseline measures administered prior to randomization. The Colorado Multiple Institutional Review Board approved the study (www.ClinicalTrials.gov identifier: NCT02037568).

### Participants

Participants were recruited between March 1st, 2014 and 4 November 4th, 2016 during routine transplant prescreening. Recruitment occurred at a community-based transplant program (n = 98) and a university‐based cancer center (n = 61). Eligibility criteria included (1) Allo‐HSCT patient and their primary caregiver both agreed to participate, (2) spoke/read English, (3) telephone access, and (4) ≥ 18 years old. Exclusion criteria included 1) uncontrolled psychiatric disorder in past 18 months. Caregivers were defined as the individual in the patient's life primarily responsible for care posttransplant, emotionally invested in the patient, and responsible for major care decisions.

### Intervention

PEPRR originated from a stress management intervention for medically ill patients [21] that was modified for caregivers originating using a cognitive behavioral framework [17]. The PEPPR intervention was delivered by three master's level social workers for eight, 60‐minute sessions during the 100-day posttransplant period. Additional details regarding the intervention are available elsewhere [17]. The first PEPPR session began 17.4 days (95% CI: 10.3–24.5) after transplant. Weekly sessions typically occurred for the first four weeks and then every other week. For the eTAU control group, caregivers were emailed all sections from the PEPRR workbook.

### Measures

The parent study examined a combined distress composite, combining measures of depression, anxiety and perceived stress [22]. In the current analyses, we examined these mood outcomes in caregivers and patients that are particularly salient in HSCT. Depression is observed to be a particularly meaningful outcome in its relationship to caregiver burden [23] and sensitive to change during the course of HSCT [15]. Depression and quality of life (QOL; compared to anxiety, fatigue or symptom burden) may also be most responsive to supportive interventions in patients and caregivers undergoing HSCT [24, 25]. Patient QOL was selected given its importance to long-term clinical outcomes in HSCT [26].

### Anxiety

Symptoms of caregiver anxiety were measured using the Speilberger State-Trait inventory of Anxiety [27]. and is validated in both healthy participants [28] and with caregivers of patients with serious illness [29]. Higher scores indicate greater anxiety. This 40-item scale has good internal consistency in our study with an alpha of 0.95 across all 4 timepoints.

### Depression

To measure caregiver depressive symptoms, we used the Center for Disease Control-Depression scale (CESD). The 20-item scale has been validated in cancer populations and in caregivers specifically, with higher CESD scores reflecting greater depression, ranging from 0 to 60. Similar to studies in the general population [30], internal reliability was adequate across all timepoints in our study (baseline *α* = 0.71; 6 weeks *α* = 0.61; 3 months *α* = 0.72; 6 months *α* = 0.78).

### Patient quality of life

To measure patient QOL, the Functional Assessment of Cancer Therapy Scale (FACT-BMT) was used. The FACT is a widely used instrument and is a comprehensive measure of QOL in HSCT [31]. Scores from 47 items combine into one continuous scale in which higher scores indicate better QOL. The questions are anchored to the past 7 days in which scores are normed and scored with possible ranges from 0 to 148. Internal reliability in the current sample was good across all 4 timepoints (baseline *α* = 0.87; 6 weeks *α* = 0.92; 3 months *α* = 0.92; 6 months *α* = 0.95).

### Statistical approach

First, we examined all descriptive statistics and bivariate correlations between study variables. Guided by the Actor Partner Interdependence Modeling framework (APIM) [32], we then tested dyadic interdependence over time with structural equation models (SEM). This approach allows for simultaneous estimation of an individuals’ QOL on their subsequent QOL ratings (“actor” effects) while controlling for other estimates in the model, in addition to testing how one member of the dyad’s QOL impacts the other member’s depression/anxiety (“partner” effects). As such, one can garner the unique contribution of each effect. Models were evaluated through consideration of model fit statistics and parameter estimates; specifically, CFI scores of > 0.90 and scores of < 0.05 indicating good model fit for RMSEA and SRMR [33]. Chi-square difference tests compared constrained and unconstrained models to test differences in parameter estimates between treatment groups. Missing data was handled by full information maximum likelihood (FIML) [34]. SPSS [35] was used for descriptive and linear models and Mplus [36] was used for all SEMs.

We examined two different models of patient and caregivers’ well-being over time: (1) a model of patient QOL and caregiver anxiety, (2) a model of patient QOL and caregiver depression. In both models, constructs were measured at the four study time points of baseline, 6 weeks, 3-months and 6-months post-transplant admission. For our primary research question of whether dyadic associations differed between the PEPPR and eTAU groups, we tested multi-group models in which all parameters were freely estimated in each group and then all parameters were constrained to be equal between groups. The fit between models was compared using chi-square difference tests. Preliminary multiple groups SEM indicated no significant differences in the fit of a constrained vs. unconstrained models, therefore we report on the more parsimonious, constrained model. Although the model constrains unstandardized estimates to be equal across groups, the standardization procedure is done separately within each group. For ease of interpretation of the size of effects, the standardized estimate from each group for all effects are presented.

## Results

Allo‐HSCT patient/caregiver dyads (n = 407) were approached at the two participating hospitals and 331 dyads met eligibility criteria. One hundred and fifty-four patient and caregivers participated in this study and provided baseline data for analyses. As previously reported [18], there were no differences on baseline demographics between those assigned to PEPPR as compared to eTAU. Greater caregiver age was also significantly related to lower caregiver depression at baseline (*r* = − 0.22; *p* = 0.006) and younger patient age was related to worse patient QOL (*r* = 0.17; *p* = 0.031). Accordingly, all models accounted for baseline age.

### Patient quality of life and caregiver anxiety

First, we examined bivariate correlations across all timepoints, which are displayed in Table 1. Higher patient QOL was significantly correlated with lower caregiver anxiety at baseline (*r* = − 0.31; *p* = 0.00028) and at 6 weeks post-admission (*r* = − 0.29; *p* = 0.007), but not at subsequent timepoints. To address the assumption in longitudinal models that data is missing at random, we conducted a series of univariate t-tests according to Enders [34] in which those participants who provided data at the final timepoint were compared to those lost at the final time point across our three primary constructs of interest, measured at baseline. We first created a variable that codes whether the caregiver was retained at the last time point or not. Then, we ran separate t-tests with this variable as the grouping variable and caregiver depression, anxiety or patient quality of life at baseline as the DV. These analyses suggested there were no significant differences in missingness in those retained and those lost to follow-up across caregiver depression (*p* = 0.797), caregiver anxiety (*p* = 0.098) and patient quality of life (*p* = 0.612).

**Table 1:** Demographic characteristics of patients and caregivers undergoing stem cell transplantation

	Caregivers^a^				Patients^c^			
	No. (%) of sample				No. (%) of sample			
	$${\overline{\text{X}}}$$	eTAU	PEPRR		$${\overline{\text{X}}}$$	eTAU	PEPRR	
Characteristics	(n = 154)	(n = 80)	(n = 74)	Significance^b^	(n = 154)	(n = 80)	(n = 74)	Significance^d^
Age, mean (CI), y	54.0 (51.9, 56.2)	54.6 (51.9, 57.4)	53.4 (50.0, 56.8)	*p* = 0.56	53.2 (50.9, 55.5)	54.3 (51.1, 57.5)	52.1 (48.6, 55.5)	*p* = 0.35
*Sex, number (%)*								
Female	123 (79.9)	66 (82.5)	57 (77.0)		55 (35.7)	28 (35.0)	27 (36.5)	
Male	31 (20.1)	14 (17.5)	17 (23.0)	*p* = 0.42	99 (64.3)	52 (65.0)	47 (63.5)	*p* = 0.85
*Race, number (%)*								
White	128 (83.1)	67 (83.8)	61 (82.4)		123 (79.9)	59 (73.8)	64 (86.5)	
Other				*p* = 0.68				*p* = 0.69
American Indian or Alaska Native	1 (0.6)	1 (1.3)	-		2 (1.3)	2 (2.5)	-	
Black or African-American	2 (1.3)	-	2 (2.7)		1 (0.6)	-	1 (1.4)	
More than one race	6 (3.9)	3 (3.8)	3 (4.1)		4 (2.6)	3 (3.8)	1 (1.4)	
Another group not listed	6 (3.9)	3 (3.8)	3 (4.1)		6 (3.9)	2 (2.5)	4 (5.4)	
*Ethnicity, number (%)*								
Hispanic or Latino	14 (9.1)	8 (10.0)	6 (8.1)		12 (7.8)	8 (10.0)	4 (5.4)	
Non-Hispanic or Latino	127 (82.5)	63 (78.8)	64 (86.5)	*p* = 0.59	118 (76.6)	55 (68.8)	63 (85.1)	*p* = 0.23^e^
Education								
College graduate or above	74 (48.1)	35 (43.8)	39 (52.7)	*p* = 0.46	74 (48.1)	38 (47.5)	36 (48.6)	*p* = 0.61
*Annual income, $ (%)*								
< 25,000	49 (31.8)	29 (36.3)	20 (27.0)		43 (27.9)	20 (25.0)	23 (31.1)	
25,000–44,999	27 (17.5)	14 (17.5)	13 (17.6)		28 (18.2)	17 (21.3)	11 (14.9)	
45,000–64,999	30 (19.5)	12 (15.0)	18 (24.3)		19 (12.3)	9 (11.3)	10 (13.5)	
> 65,000	29 (18.8)	13 (16.3)	16 (21.6)	*p* = 0.36	30 (19.5)	14 (17.5)	16 (21.6)	*p* = 0.64
*Relationship, number (%)*								
Spouse/partner	100 (64.9)	53 (66.3)	47 (63.5)					
Parent	20 (13.0)	11 (13.8)	9 (12.2)					
Other	29 (18.8)	11 (13.8)	18 (24.3)	*p* = 0.33				
*Patient Diagnosis*^*f*^*, number (%)*								
Leukemia					93 (60.4)	50 (62.5)	43 (58.1)	
Lymphoma					20 (13.0)	11 (13.8)	9 (12.2)	
MDS/MPS					35 (22.7)	16 (20.0)	19 (25.7)	
Other (MM, SAA)					6 (3.9)	3 (3.8)	3 (4.1)	*p* = 0.51^g^

^a^Caregiver information was not available for eTAU for the following variables: race (n = 6), ethnicity (n = 9), education (n = 5), annual income (n = 12), and relationship (n = 5). PEPRR for the following variables: race (n = 5), ethnicity (n = 4), and annual income (n = 7)

^b^Significance from independent t-test or Pearson's Chi-square test as appropriate

^c^Patient information was not available for eTAU for the following variables: race (n = 14), ethnicity (n = 17), education (n = 11), and annual income (n = 20). PEPRR for the following variables: race (n = 4), ethnicity (n = 7), education (n = 3), and annual income (n = 14)

^d^Significance from independent t-test or Pearson's Chi-square test as appropriate

^e^Significance from Fisher's Exact Test

^f^MDS, myelodysplastic syndrome; MPS, myeloproliferative syndrome; MM, multiple myeloma; SAA, severe aplastic anemia

^g^Significance from Mann Whitney U Test

**Fig. 1:**
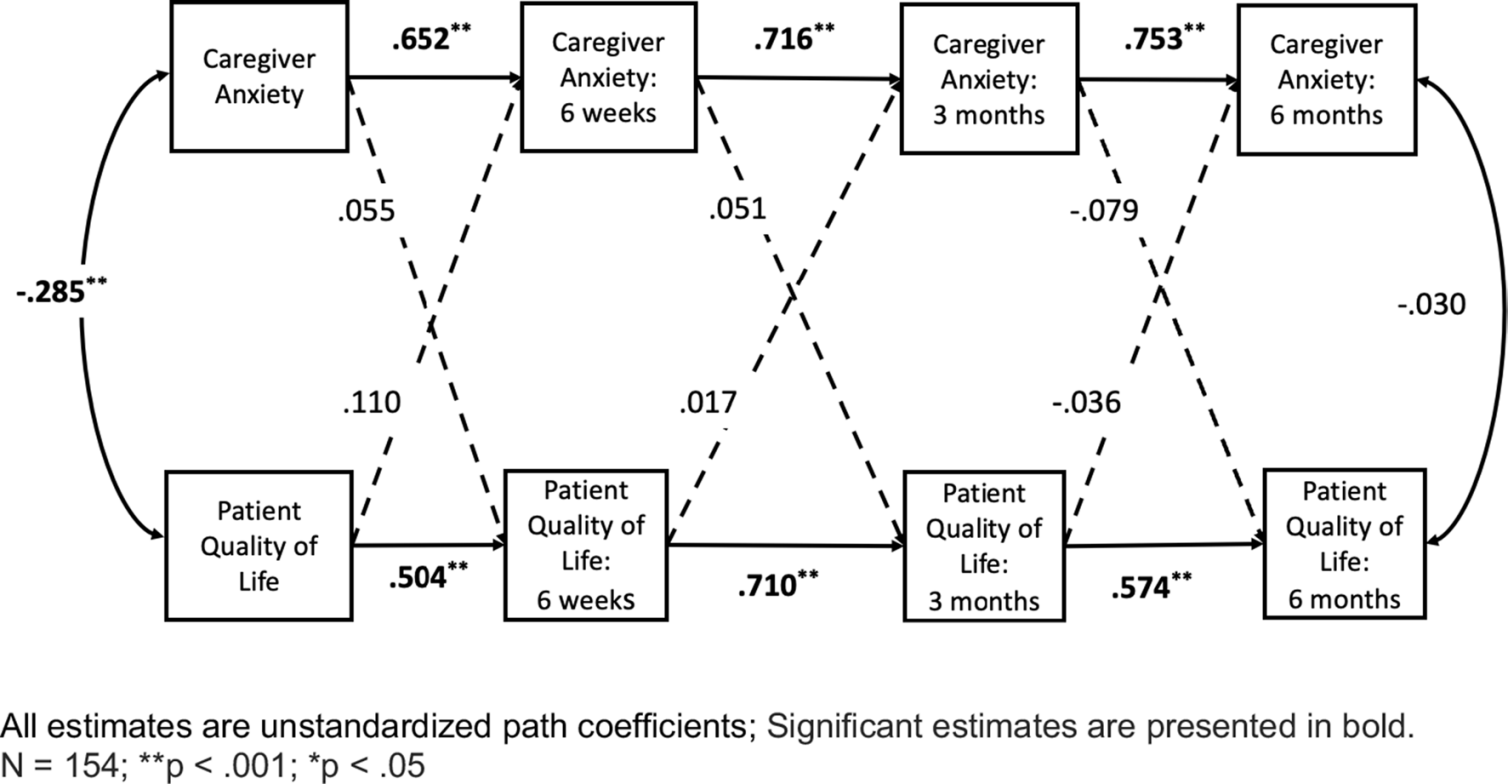
APIM model of caregiver anxiety and patient quality of life across the course of hematopoietic stem cell transplant

Next, within the SEM model of patient QOL and caregiver anxiety, all actor effects were significant (all *p’s* < 0.05). This means that prior patient QOL was a significant predictor of subsequent patient QOL at each of the three follow-up time points. Similarly, caregiver anxiety was a significant predictor of subsequent caregiver anxiety at each of the three follow-up time points (Fig. 1).

### Partner effects

No significant partner effects were supported meaning that caregiver anxiety did not predict subsequent patient QOL when controlling for prior patient QOL. Similarly, patient QOL did not predict subsequent caregiver anxiety when controlling for prior caregiver anxiety. Importantly, as suggested in bivariate comparison, a significant correlation was shown between baseline caregiver anxiety and patient QOL. A significant relationship was not found for the final time point (Fig. 1).

### Patient quality of life and caregiver depression

First, bivariate relationships demonstrated that higher patient QOL was significantly related to lower caregiver depression at all timepoints, except for at the 3-month timepoint (*r* = − 0.048, *p* = 0.690). Specifically, across the course of stem cell transplant, the relationship between patient QOL and caregiver depression changed, such that the two variables were significantly related at baseline (***r*** = − 0.32; *p* = 0.000), 6 weeks (*r* = − 0.22; *p* = 0.044), and the 6-month timepoint (***r*** = − 0.34; *p* = 0.009; Table 2).

**Table 2:** Bivariate correlations among patient and caregiver well-being over time

Patient quality of life—Baseline (M = 97.94; SD = 18.25)	− .32**	− .12	.048	− .16	− .31**	− .13	− .10	− .15
Patient quality of life—6 weeks (*M* = 96.33; SD = 19.39)	− .042	− .22*	.12	− .11	− .15	− .29*	− .15	− .15
Patient quality of life—3 months (M = 101.43; SD = 19.09)	.029	− .044	− .048	− .19	− .11	− .16	− .20	− .17
Patient quality of life—6 months (M = 101.49; SD = 21.89)	− .23	− .14	− .26	− .34**	− .22	− .11	− .33**	− .24
	Caregiver Depression—Baseline (M = 20.29; SD = 6.61)	Caregiver Depression—6 weeks (M = 19.26; SD = 5.58)	Caregiver Depression—3 months (M = 19.0; SD = 6.40)	Caregiver Depression—6 months (M = 20.06; SD = 7.20)	Caregiver Anxiety—Baseline (M = 40.59; SD = 12.88)	Caregiver Anxiety—6 weeks (M = 38.98; SD = 12.21)	Caregiver Anxiety—3 months (M = 38.41; SD = 12.64)	Caregiver Anxiety—6 months (M = 37.65; SD = 12.98)

N = 154; **p < .001; *p < .05

Missingness was as follows:

Caregiver CESD baseline: 0% missing; 6 weeks: 22.7%; 3 months: 37.6%; 6 months: 46.1%

STAI baseline: 0% missing; 6 weeks: 21.4%; 3 months: 37.0%; 6 months: 45.5%

Patient FACT baseline: 0% missing; 6 weeks: 30.5%; 3 months: 38.3%; 6 months: 50.0%

Cronbach’s alpha across each timepoint were as follows:

Caregiver CESD baseline: .71; 6 weeks: .61; 3 months: .72; 6 months: .78

STAI baseline: 95; 6 weeks: .95; 3 months: .95; 6 months: .95

Patient FACT baseline: .87; 6 weeks: .92; 3 months: .92; 6 months: .95

Next, we estimated longitudinal SEM models as described above. Across all timepoints, significant actor effects emerged. This indicated that patients’ QOL predicted subsequent ratings of their own quality of life at latter time points (all *p’s* < 0.05). Similarly, all caregiver ratings of their own depression were significantly related to subsequent ratings of their own depression at later time points (all *p’s* < 0.05).

### Partner effects

**Fig. 2:**
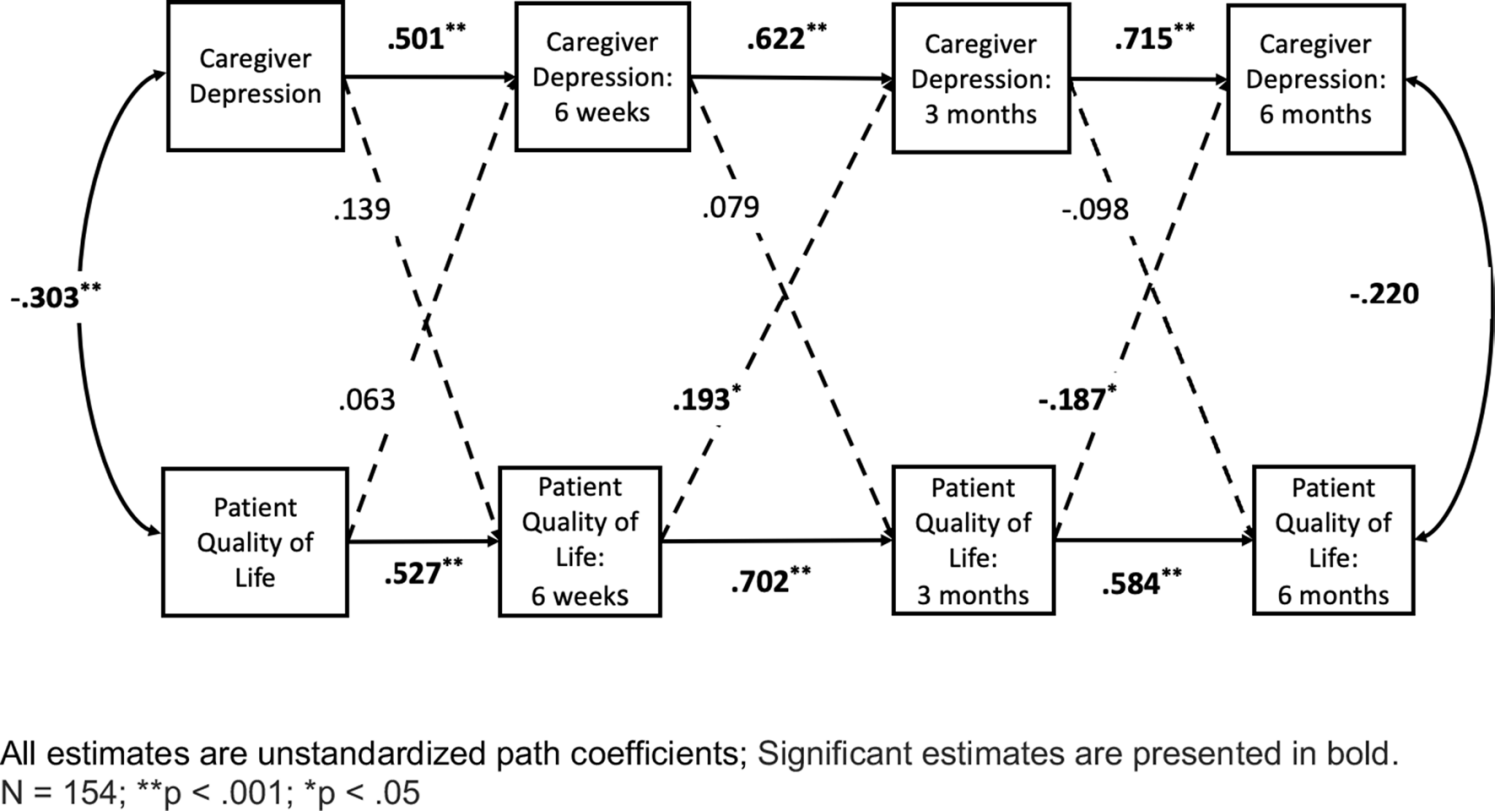
APIM model of caregiver depression and patient quality of life across the course of hematopoietic stem cell transplant. All estimates are unstandardized path coefficients; Significant estimates are presented in bold. N = 154; **p < .001; *p < .05

Two significant partner effects emerged in our analyses. First, worse patient QOL at 6 weeks was significantly related to lower caregiver depression at 3 months following transplant (***B* = 0.193**; *p* = 0.028). Second, this relationship also changed over time, such that worse patient QOL at 3 months was significantly related to more caregiver depression at 6 months (***B* = **− **0.187**; *p* = 0.001; Fig. 2).

### Exploratory analyses

Because the first partner effect was in the unanticipated direction (worse patient QOL related to lower depression at the subsequent timepoints), we ran a series of follow-up regression models to isolate this effect and potentially explain this finding. We considered two patient variables as potential moderators: days of patient hospitalization and number of readmissions to the hospital. Although the number of readmissions was not related to change in caregiver depression between 6 weeks and 3 months, greater days in the hospital approached significance in its relation to greater caregiver depression at 3 months (*B* = 0.191; *p* = 0.093), after controlling for caregiver depression and patient QOL at 6 weeks. This suggests that length of the hospital stay may be related to the interdependence of patient QOL and caregiver depression at 3 months post-HSCT.

## Discussion

Caregivers are critical to patients during the cancer journey, particularly in HSCT. There is emerging data highlighting that patient and caregiver well-being is often interdependent [20], but may change over time [37, 38]. The current study aimed to examine the interdependence of patients’ QOL and caregivers’ anxiety and depression. Overall, it is noteworthy that the data fit one parsimonious model as opposed to a multigroup SEM for the PEPPR and eTAU groups as originally hypothesized suggesting similar associations between dyads and across time in these two groups. In the full sample, we observed significant actor effects over the course of HSCT (patients’ QOL scores predicting their subsequent QOL and caregivers’ depression predicting their own subsequent depression). Additionally, we detected somewhat surprising partner effects, suggesting the interdependence changes over time; namely reverses direction such that if a patient has worse QOL at 6 weeks, their caregiver will be doing better (lower depression) at the 3-months. Finally, in visual comparison across the models examining caregivers’ anxiety and depression, there is some suggestion of greater interdependence for patient QOL to caregiver depression, than for patient QOL to caregiver anxiety across HSCT.

It is first worth commenting on the finding that the data did not fit a multigroup model as originally hypothesized. There are several reasons why the constrained model was retained over the multigroup model. We may have been underpowered to detect group differences with multiple estimated parameters, a common concern in longitudinal SEM’s [39]. Further, while the PEPPR intervention demonstrated a significant effect on caregiver distress [18], this effect may not be robust to extend to patient QOL, or dyads’ interdependence over time. As demonstrated in exploratory regression models, a number of medical factors likely impact QOL (length of hospitalization) and may overshadow any dyadic relationships of well-being. Future studies will benefit from more repeated measures of patients and caregivers, potentially harnessing technologies to track changes over more granular periods of time [40] and closely monitoring how changes in patient status relate interdependence.

The dyadic changes that we detected over the course of transplant, while small in effect, are intriguing for several reasons. First, the significant relationship that we expected between FC mood and patient QOL at baseline based on prior work [20] continued at 6 weeks but was no longer significant at 3 months. This may relate to patient and caregivers’ interrelatedness changing as care transitions to an outpatient setting in which more care is provided by the medical team and, potentially, relieve the caregiver or impose additional details of patient care that caregivers feel they need to observe. Second, we detected an unanticipated change in the direction of the relationship between patient QOL at 6 weeks and caregivers’ depression at 3-months in that caregivers were less depressed at 3 months when their patient had poorer QOL at 6-weeks post-HSCT. Changes in patients’ medical treatment—experienced by both members of the dyad—may also explain changing interdependence. We attempted to explore this hypothesis by looking at the impact of hospitalized days as predictors of in the regressions suggesting that the greater number of days that patients were in the hospital approached significance as a predictor of greater depression at 3-months post-transplant. Future research can build on these initial findings by closely monitoring changes in patients’ medical status.

The primary analyses with the current data showed the intervention improved distress overall, with slightly greater effect sizes for anxiety than depression. The current analyses explored dyadic effects within these constructs and although the associations among patient quality of life and caregiver mental health did not differ between the intervention and control conditions, dyadic interdependence may still be important to consider when designing and evaluating interventions. This work provides evidence of dyadic interdependence which may affect the success, or lack thereof, of an intervention. For instance, there is evidence of strong mutual influences on health between close relationship partners [41]. Physical health problems often negatively impact mental health of partners in close relationships; at the same time, interdependence can be leveraged to improve intervention effectiveness [41]. In addressing health behavior, interventions targeting partners can help patients make behavior change, by reinforcing positive behaviors [42]. This model has been extended to how shared health behaviors and emotional experience can impact biobehavioral pathways that impact health and disease [43] and our group has demonstrated how the shared behavior withing couples (e.g., sleep quality) can impact immune reconstitution in HSCT [44]. Overall, dyadic interdependence remains an important consideration in designing and testing interventions, particularly in HSCT.

The current findings should be qualified with several limitations. First, the patients and caregivers recruited were fairly homogenous, with the majority of caregivers being white females and patient-caregiver dyads being spousal. Future research should seek a more diverse demographic sampling to increase generalizability and examine whether similar patterns of interdependence are observed in other patient-caregiver relationships (e.g., parent–child dyads). Additionally, the current analyses represent secondary data analyses and the original study may not have been powered to detect dyadic relationships over time, as multigroup SEMs often require hundreds of participants [39]. Larger samples will allow for additional mediation or moderation models to further explore dyadic relationships [45], or potentially, different mechanisms [46] of intervention effects. Finally, we had a relatively high rate of dropout at the later timepoints in this study (> 40% at the 6-month timepoint). While we attempted to address the assumptions of data being missing at random [34] and our findings did not suggest significant differences between those providing data at the final 6 months timepoint and those lost to follow-up, it is possible that our findings would only generalize to a population of patients and caregivers that are willing and able to sustain participation in a longitudinal study.

## Conclusions

This study highlights the interrelated, yet nuanced, patient and caregiver relationship during the course of stem cell transplantation. These results highlight the need for not only additional support for the patient, but also the caregiver and possibly the closely connected dyad. While future work may identify how these change in the context of the intervention, our findings imply that the association between patient QOL and partner depression may differ across the course of transplant and interventions that seek to improve patient-caregiver well-being should remain mindful of these differences when study dyadic relationships. Future research should explore these relationships and include longer-term follow-up to identify when, and how, to better support patients and their closely connected caregivers.

## Data Availability

The datasets used and/or analysed during the current study are available from the corresponding author on reasonable request.
